# Surgical treatment of lower urinary tract symptoms secondary to benign prostatic obstruction: an analysis and meta-synthesis of available guidelines

**DOI:** 10.1186/s12894-025-01788-6

**Published:** 2025-04-24

**Authors:** Laurenz S. Matter, Orlando Burkhardt, Pavel Lyatoshinsky, Jennifer Blarer, Roland Seiler, Dominik Abt

**Affiliations:** 1 Department of Urology, Klinik für Urologie, Hospital Center Biel, Spitalzentrum Biel, Vogelsang 84, Biel/Bienne, 2501 Switzerland; 2 University of Bern, Bern, Switzerland; 3 Department of Urology, Cantonal Hospital of St. Gallen, St. Gallen, Switzerland; 4 Universitätsklinik für Urologie, Inselspital Bern, Bern University Hospital, Bern, Switzerland

**Keywords:** Benign prostate obstruction, Lower urinary tract symptoms, Minimally invasive treatments, Guidelines

## Abstract

**Purpose:**

The increase in minimally invasive treatments (MITs) for lower urinary tract symptoms secondary to benign prostatic obstruction (LUTS/BPO) has diversified surgical options, often outpacing solid evidence. The variety of available treatments, while beneficial, can confound physicians. Clinical guidelines provide direction but often differ due to varied evidence interpretation.

**Methods:**

We have analyzed the available guidelines on the surgical treatment of LUTS/BPO updated within the last three years, focusing on those offering specific procedural recommendations. We compared recommendations, analyzed discrepancies, and developed a consensus algorithm that incorporated all pertinent advice.

**Results and limitations:**

Out of 14 guidelines, four met the inclusion criteria. Major challenges were inconsistent nomenclature and a lack of clear recommendations, especially for newer procedures such as Temporary Implantable Nitinol Device (iTIND™), Prostate Artery Embolization (PAE), Robotic Assisted Simple Prostatectomy (RASP), and Water Vapor Thermal Therapy (Rezūm™). Despite these issues, a consensus algorithm could be synthesized.

**Conclusions and clinical implications:**

Guidelines for the treatment of LUTS/BPO present a disparate picture, with consensus mostly on older, well-established procedures due to substantial evidence. Newer interventions display significant variation in guideline recommendations and evidence interpretation. The consensus algorithm created from current guidelines offers a synthesized overview of recommendations, underscoring the need for standardized evidence criteria for guideline recommendations. Our work emphasizes the evolving complexity in LUTS/BPO management, aiming to aid urologists in decision-making and patient counseling by providing a clear and comprehensive tool.

**Supplementary Information:**

The online version contains supplementary material available at 10.1186/s12894-025-01788-6.

## Introduction

Lower urinary tract symptoms supposed secondary to benign prostatic obstruction (LUTS/BPO) are among the most prevalent conditions in older males [1]. Surgical treatment is usually recommended if pharmacotherapy fails, is not well tolerated, or when BPO-related complications arise. Surgical options can also be used at an earlier stage after a thorough joint decision has been made. As established surgical therapies have drawbacks and the threshold for surgical treatment seems to have become much lower with the implementation of minimally invasive treatments (MITs), LUTS/BPO surgery has become an economically significant area [2]. Consequently, novel therapeutic devices and procedures are being developed at an unprecedented rate.

The rapid adoption of new surgical techniques is influenced by a range of factors, including patient demand, competitive healthcare dynamics, and regulatory pathways that are often less stringent than those applied to pharmaceuticals. Consequently, some interventions may enter clinical use before a comprehensive evidence base is available [3, 4]. A comprehensive understanding of the array of procedures, including their benefits and risks, safety profiles, and ideal indications, is essential for successful, individualized treatment. However, for physicians not deeply specialized in the field, staying abreast of these developments can be challenging [5]. Clinical guidelines provide critical support in this context.

Nonetheless, guidelines are not without their limitations. The required level of evidence for recommending novel treatments is not uniformly defined [3]. Furthermore, while guidelines should rely on robust evidence, they also need to remain relevant and not withhold potentially beneficial novel treatments from patients. Consequently, the same body of evidence might receive varying interpretations by different guideline bodies.

The objective of this study was to analyze existing guidelines for the surgical treatment of LUTS/BPO, compare their recommendations for various surgical methods and treatment scenarios, and assess where further data could unify recommendations where discrepancies exist.

## Methods

We conducted a structured and comprehensive search for guidelines on the surgical treatment of LUTS/BPO. To ensure broad international coverage, we systematically screened the websites of all national societies listed as members of the Société Internationale d’Urologie (SIU). This approach was chosen because many relevant guidelines are not published in peer-reviewed journals and may only be accessible via professional society websites. A conventional PRISMA-based systematic review would not have identified these documents and was therefore not suitable for our research objective.

Our methodology aligns with the approach described by Zumstein et al. in a prior BMC Urology publication [6], which used a similar society-based strategy to identify and compare urological guidelines. In addition, we conducted a targeted online search to capture any recent guidelines not listed by SIU members but relevant to our topic.

Eligibility for further analysis was determined by the following criteria: (I) surgical treatment recommendations for LUTS/BPO, (II) guidelines updated within the last three years as of 15th April 2024, (III) current versions not considered obsolete, and (IV) guidelines providing not just general surgical recommendations but also those discriminating between treatment scenarios based on patient-specific factors, such as prostate volume and anatomy (i.e. presence of a middle lobe).

**Fig. 1:**
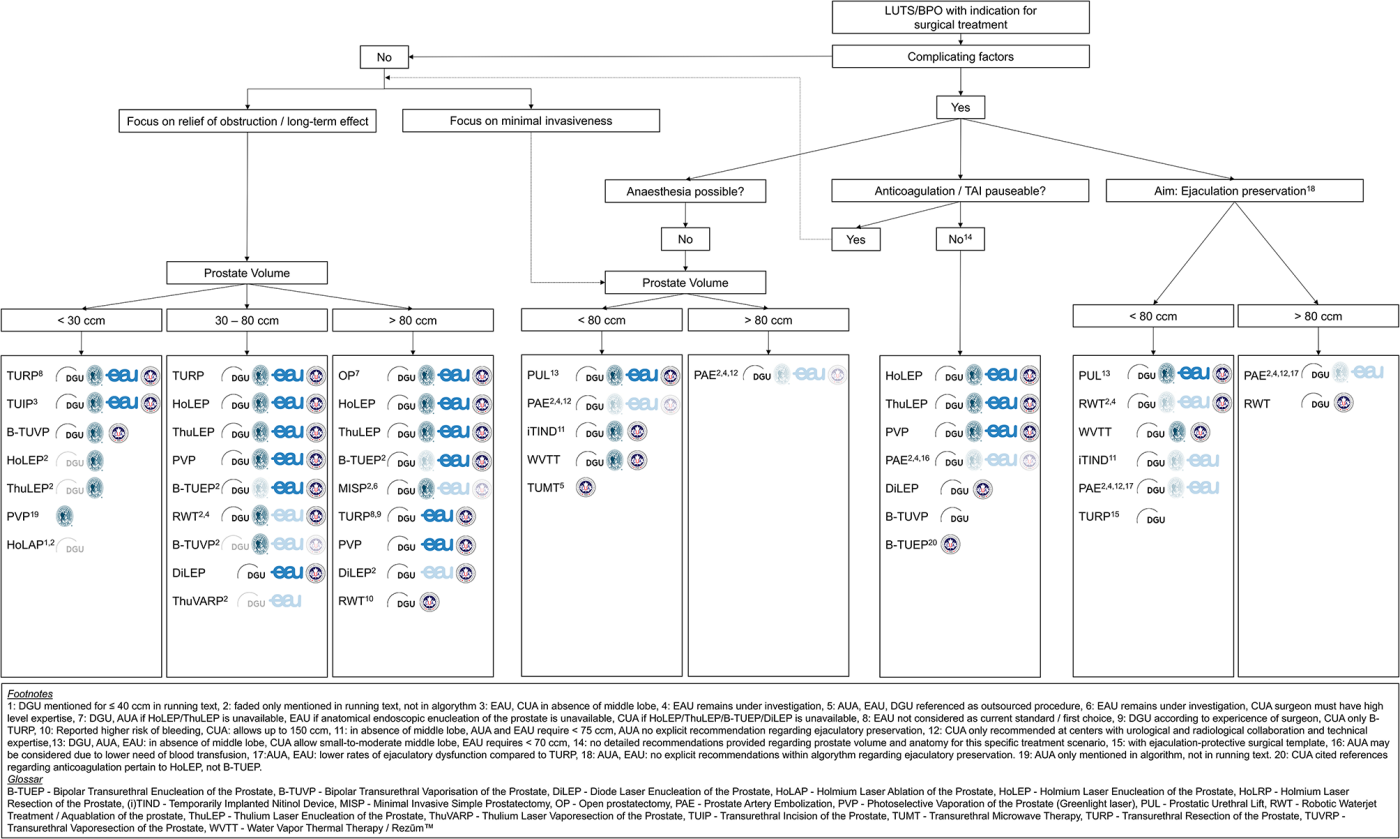
Consensus Algorithm

The identified guidelines were examined for their recency, comprehensiveness, level of detail, procedural recommendations, and statements on specific treatment scenarios, indications, and restrictions. The assessments of written and graphical recommendations were independently conducted by four authors (DA, LM, OB, PL), with discrepancies discussed until consensus was reached. A synthesis of continuous texts and illustrations from the guidelines resulted in a general algorithm (Fig. 1), as previously described [6], which helped to highlight and address any discrepancies identified.

## Results

### Guideline and comparison

Among the 138 SIU member states, we identified 14 separate urological guidelines on the surgical treatment of LUTS/BPO (Table 1). Approximately 44% of these were also published in peer-reviewed journals. The guidelines clearly varied in length, ranging from 14 to 355 pages. The Dutch Society for Urology (NVU) [7], the Singapore Urological Association (SUA) [8], and the Urological Society of India (USI) [9] included references to the European Association of Urology (EAU) in their guidelines. The guidelines from the Colombian Society of Urology (SCU) [10] referred to the American Urological Association (AUA).

**Table 1:** Identified guidelines on the surgical treatment of LUTS / BPO

Abv.	Society Name	Country / Continent	Last Update	Extent (pages)^1^	Algorithm surgical BPO treatment	IF^2^	Reference to Guidelines of	Citations	Reason for exclusion
AUA	American Urological Association	USA	14.09.2023	76	Yes	7.641	-	183	na (included)
AURO	Association of Italian Urologists	Italy	01.01.2004	44	Yes	-	-	-	outdated
EAU	European Association of Urology	Europe	01.04.2024	124	Yes	-	-	-	na (included)
CUA	Canadian Urological Association	Canada	11.04.2022	12	Yes	2.052	-	101	na (included)
DGU	German Society of Urology	Germany	28.02.2023	247	Yes	-	-	-	na (included)
IAUI	Indonesian Urology Association	Indonesia	01.01.2021	42	Yes	-	-	-	language
NICE	National Institute of Health and Care Excellence	United Kingdom	01.06.2015	355	No	5.296	-	101	outdated
NVU	Dutch Society for Urology	Netherlands	01.01.2017	230	No	-	EAU	-	outdated
JUA	Japanese Urological Association	Japan	23.05.2017	14	Yes	1.844	-	118	outdated
KUA	Korean Urological Association	Korea	11.01.2016	16	No	1.902	-	45	outdated
SAU	Argentinian Urological Association	Argentina	01.01.2012	76	No	-	-	-	outdated
SCU	Colombian Society of Urology	Columbia	01.01.2021	60	Yes	-	AUA	-	language
SUA	Singapore Urological Association	Singapore	01.06.2018	8	No	1.081	EAU	11	outdated
USI	Urological Society of India	India	17.07.2019	66	No	-	EAU	-	outdated

(^1^ including non-surgical content ^2^IF: impact factor, if (additionally) published in journal)

We excluded eight guidelines from further analysis due to their publication date being outside the three-year threshold as of 15th April 2024. Two additional guidelines were not included due to language barriers (Table 1), underscoring the challenge of accessibility and the potential for linguistic limitations to impact guideline dissemination.

Subsequently, the following guidelines were integrated into further analysis: American Urological Association (AUA) [11], the European Association of Urology (EAU) [12], the Canadian Urological Association (CUA) [13], and the German Society of Urology (DGU) [14].

### Challenges and discrepancies in analysis of identified guidelines

The comparative analysis revealed several discrepancies, particularly in structural concepts and procedural nomenclature. For instance, the same surgical procedure might be listed under different names across guidelines, complicating the comparison. This was particularly evident in the classification of techniques like vaporization, vaporesection, and enucleation and the inconsistent usage of terms related to various energy sources.

Furthermore, differing categorizations and groupings of procedures and broad, general recommendations for groups of interventions were found. For example, the CUA [13] does not provide individualized recommendations for different enucleation techniques, instead they are summarized with the collective term Anatomic Endoscopic Enucleation of the Prostate (AEEP), without specific distinctions in the recommendations provided.

The most significant differences in specific treatment recommendations were noted for newer or minimally invasive procedures such as iTIND™, Prostate Artery Embolization (PAE), Robotic Assisted Simple Prostatectomy (RASP), and Rezūm™ (Table 2).

**Table 2:** Statements of the examined guidelines on currently frequently employed and debated procedures

Treatment	AUA	CUA	DGU^2^	EAU	DoC^1^
**iTIND™**	TIPD may be offered as a treatment option for patients with LUTS/BPH provided prostate volume is between 25 and 75 g and lack of obstructive median lobe. ***(LoE: Grade C)***	We recommend that iTIND™ may be offered to men with LUTS interested in preserving ejaculatory function, with prostates 30–80 cc. Patients should be made aware of the higher retreatment rate at 3 years. ***(LoE: Grade C)***	The temporary implantable nitinol basket may serve as an alternative therapy for benign prostatic syndrome in prostates with a volume of up to 75 cubic centimeters and without an endovesical median lobe. Particularly for patients desiring the preservation of ejaculation, therapy with a temporarily implanted nitinol basket can be offered. ***(LoE: expert opinion)***	Randomized controlled trials comparing iTIND™ to a reference technique are ongoing. ***(LoE: under investigation)***	intermediate
**PAE**	PAE may be offered for the treatment of LUTS/BPH. PAE should be performed by clinicians trained in this interventional radiology procedure following a discussion of the potential risks and benefits. ***(LoE: Grade C)***	At centers with urological and radiological collaboration and technical expertise, highly selected, well-informed patients may be offered PAE if they wish to consider an alternative treatment option. Patients should be informed of lack of long-term durability ***(LoE: Grade C)***	Prostate artery embolization should be considered for patients with benign prostatic syndrome who are suitable and willing to accept a lesser improvement of objective micturition parameters with this minimally invasive therapy. ***(LoE: Grade 1++)***	Prostatic artery embolisation (PAE) is less effective than TURP at improving symptoms and urodynamic parameters such as flow rate. ***(LoE: Grade 1a)*** Procedural time is longer for PAE compared to TURP, but blood loss, catheterisation and hospitalisation time are in favour of PAE. ***(LoE: Grade 1b)***	high
**RASP**	Open, laparoscopic, or robotic assisted prostatectomy should be considered as treatment options by clinicians, depending on their expertise with these techniques, only in patients with large to very large prostates. ***(LoE: Grade C)***	We recommend LSP or RASP as alternative surgical therapies for men with moderate-to-severe LUTS/BPS and enlarged prostate volume > 80 cc in centers where there are surgeons with high-level expertise in robotics or laparoscopy. ***(LoE: Grade B)***	The use of modern surgical techniques based on endoscopy and laparoscopy with or without robotic assistance can reduce the risk of complications such as increased blood loss or extended hospital stays, while maintaining the same effectiveness, despite a longer duration of surgery. ***(LoE: expert opinion)***	Minimal invasive simple prostatectomy is feasible in men with prostate sizes > 80 mL needing surgical treatment; however, RCTs are needed. ***(LoE: Grade 2a)***	low-intermediate
**Rezūm™**	WVTT should be considered as a treatment option for patients with LUTS/BPH provided prostate volume 30–80 g. ***(LoE: Grade C)*** WVTT may be offered as a treatment option to eligible patients who desire preservation of erectile and ejaculatory function. ***(LoE: Grade C)***	We suggest that the Rezūm™ system of convective water vapor energy ablation may be considered an alternative treatment for men with LUTS interested in preserving ejaculatory function with prostates < 80 cc, including those with a median lobe. ***(LoE: Grade C)***	The Rezūm™ procedure can be a therapeutic option for benign prostatic syndrome in patients who wish to preserve ejaculation. ***(LoE: Grade 2+)***	Randomized controlled trials against a reference technique are needed to confirm the first promising clinical results and to evaluate mid- and long-term efficacy and safety of water vapour energy treatment. ***(LoE: under investigation)***	high

(^1^Degree of concordance regarding agreement on the recommendation of the procedure, ^2^Original text was translated into English, LoE = Level of Evidence)

Evidence levels were not only provided in different classifications, making comparison considerably more difficult, but there were also significant differences in the recommendation of these new procedures. For example, while general caution was advised for Rezūm™, divergent opinions were especially marked for PAE, which the DGU and EAU supported, while the AUA and CUA reserved their recommendations pending further investigation.

Thus, it becomes apparent that when the body of evidence is of lower quality, the recommendations and their strength can vary widely (Table 2).

### Synthesis of a consensus algorithm

After addressing the aforementioned discrepancies, a consensus algorithm synthesizing the recommendations from the four included guidelines was developed (Fig. 1). Thus, all guideline recommendations were merged into a unified, detailed flow-chart.

Divergent categorizations and recommendations were reconciled using additional footnotes. For example, variations in size classification were observed, with the AUA [11] categorizing prostate size not only up to > 80 cc but also from > 80-150 cc and beyond. In addition, there were a few procedures that did not fit the size classifications. For instance, the EAU only approves the use of Prostatic Urethral Lift (PUL) for prostatic glands not exceeding a volume of 70 cc. Furthermore, both the EAU and the AUA limit their recommendation for iTIND™ to prostate sizes under 75 cc.

Further cross-references were introduced to clarify procedures labeled as ‘under investigation’ by specific guidelines, prerequisites like the absence of a median lobe for some treatments, and recommendations predicated on the surgeon’s expertise or a center’s capability to provide urological-radiological collaboration.

Moreover, most guidelines did not distinctly classify their recommendations to emphasize either relief of obstruction and long-term effects or minimally invasive nature as seen in the DGU model. This necessitated a significant number of cross-references in our consensus algorithm (Fig. 1) to adequately address exceptional cases.

There were also instances where guidelines lacked concrete recommendations for procedures feasible without interruption of anticoagulation, general anesthesia, or for preserving ejaculatory function, making comparisons challenging in these specific contexts. It should be emphasized that for procedures classified as aiming to preserve ejaculation, there are sparse recommendations in the guidelines. This might be due to the fact that ejaculatory function is rarely a primary study outcome and that traditional techniques, such as TURP, can also be performed with an ejaculatory preserving approach.

Inconsistencies were occasionally found between the narrative recommendations and those depicted in the guideline algorithms. For example, the EAU textually recommends transurethral resection of the prostate (TURP) only for prostate sizes of 30-80 cc, but the visual algorithm suggests TURP for prostates smaller than 30 cc.

Furthermore, recommendations were not simply binary as ‘recommended’ or ‘not recommended’; they exist on a spectrum where some procedures were preferred, differed in levels of evidence, or were provisionally endorsed despite being ‘under investigation’.

The substantial interpretive latitude meant that not all authors uniformly interpreted the recommendations, necessitating in-depth discussions and the insertion of clarifying comments and cross-references to arrive at a consensus and an intelligible algorithm (Fig. 1).

## Discussion

The multitude of guidelines for the treatment of benign prostatic obstruction underscores the complexity and diversity of this medical issue. However, many of these guidelines are either not current or lack specific recommendations for particular procedures or treatment scenarios for individual patients. The greatest consensus among guidelines was observed for the oldest and most established procedures, attributable to the robust body of evidence supported by numerous randomized controlled trials (RCTs) [15].

Particularly regarding newer and less evaluated procedures, there are relevant discrepancies in the recommendations among the analyzed guidelines. These recommendations exhibit a wide variation in the levels of evidence and, occasionally, a notable lack of consensus across the guidelines. This is particularly evident with procedures such as iTIND™, PAE, and RASP as presented in Table 2. For instance, PAE is endorsed by the DGU and the EAU, backed by a relatively substantial level of evidence, in contrast to the more conservative positions of the AUA and CUA, which assign it an evidence level of Grade C. Thus, it is anticipated that forthcoming research will substantially improve the evidence landscape, compelling corresponding updates to the recommendations.

Several factors contribute to the observed discrepancies between guidelines. These differences may be due to the guidelines being drafted at different times with varied underlying bodies of evidence. Additionally, the interpretation of available evidence can differ significantly, particularly when the evidence base is limited. Moreover, not all current guidelines have clearly defined rules specifying which exact level of evidence is necessary, including aspects such as study size, treatment effects, and duration of follow-up, which can lead to inconsistencies. In this context, the efforts of the EAU Guidelines should be positively emphasized. Their description of the required body of evidence and the definition of meaningful follow-up periods represents an important step towards transparency and consistency.

In addition, grouping similar surgical procedures may lead to a better overview. However, if the entire group of operations is then recommended for a very specific scenario, this recommendation may no longer be adequately covered by the available evidence. An example of this is the joint recommendation of enucleation procedures (AEEP) in the CUA guidelines for patients on anticoagulation, despite the fact that there is only evidence for HoLEP, but not for B-TUEP.

Furthermore, guidelines are criticized for having various methodological and structural issues that could influence their recommendations [16]. These include widespread conflicts of interest from sponsors that support the guidelines. Conflicts of interest, both professional and financial, are also prevalent among guideline authors and boards [17]. Such conflicts, along with industry influence on guideline panels and “panel stacking” (biased selection of panel members), can impact the integrity of the guidelines. The lack of independent peer review before guideline publication is another concern that could compromise the reliability of the recommendations [18–20].

Our study builds on the work by Enikeev et al. [21], which compared recommendations using the AGREE II tool for the AUA, EAU, and NICE guidelines. Our analysis includes updated guidelines from both the DGU and CUA and excludes outdated NICE guidelines, providing a more current and comprehensive perspective. The consensus algorithm developed through our analysis (Fig. 1) not only integrates the most accepted and recent guidelines but also offers a detailed and balanced interpretation of these guidelines, independently assessed by four authors. We want to emphasize that our presented algorithm provides a structured overview of current guideline recommendations, it is not intended as a validated clinical decision-making tool. Rather, it serves to illustrate areas of agreement and divergence between established guidelines and to support clinicians in navigating an increasingly complex therapeutic landscape. A formal validation of the algorithm would require a separate prospective study, which is beyond the scope of this work.

The limitations of our study have already been addressed and predominantly arise from inconsistent classifications and categorizations within and across various guidelines. Furthermore, despite our structured and comprehensive search strategy, it is possible that some relevant guidelines were missed, particularly if they were not listed via SIU membership or not publicly available online. This represents a potential source of selection bias. In addition, by limiting inclusion to guidelines published in English or German (Table 1), some documents in other languages may have been excluded. While this was necessary for practical reasons, it may have reduced the global representativeness of our sample.

In addition, the clarity of the consensus flow-chart may be affected by the numerous footnotes. However, given the complexities outlined above and the fact that most guidelines include only vague statement on factors such as patient-preferences, therapy under anticoagulants, preservation of ejaculatory function, such intricacies were inevitable. Therefore, further simplification would have likely led to a loss of precision.

Lastly, as our focus was on the content of the guidelines rather than their methodological rigor, we did not formally assess their development process (e.g. via AGREE II), which could have further informed the interpretation of their recommendations.

## Conclusions

In summary, a multitude of guidelines for the surgical treatment of LUTS/BPO is available. They largely agree on established therapies backed by a substantial body of evidence but also exhibit relevant discrepancies. These differences may not only indicate gaps in evidence but also the need for clearer criteria regarding the evidence required for a guideline recommendation of novel treatments. The plethora of treatment options facilitates more patient-oriented therapy. However, this also means that guidelines should pay more attention to systematic representation of patient factors, such as comorbidities, anticoagulation status, and desire to preserve ejaculation. Our consensus algorithm not only facilitates a quick overview of different scenarios but also highlights areas of agreement and discrepancy among key guidelines, aiding urologists in decision-making and patient counseling.

## Electronic Supplementary Material

Below is the link to the electronic supplementary material.

Supplementary Material 1

Supplementary Material 2

## Data Availability

All data generated or analyzed during this study are included or referred to in this published article [and its supplementary information files]. All analyzed guidelines are freely available.

## Abbreviations

AEEP: Anatomic endoscopic enucleation of the prostate
AUA: American urological association
BPO: Benign prostatic obstruction
CUA: Canadian urological association
DGU: German society of urology
DiLEP: Diode laser enucleation of the prostate
EAU: European association of urology
HoLEP: Holmium laser enucleation of the prostate
iTIND™: Temporary implantable nitinol device
LUTS: Lower urinary tract symptoms
MITs: Minimally invasive treatments
NVU: Dutch society for urology
PAE: Prostatic artery embolization
PVP: Photoselective vaporization of the prostate
RASP: Robotic assisted simple prostatectomy
RCTs: Randomized controlled trials
SIU: Société international d’urology
SUA: Singapore urological association
ThuLEP: Thulium laser enucleation of the prostate
TURP: Transurethral resection of the prostate
USI: Urological society of india
WVTT: Water vapor thermal therapy
