# Supplementary figures and images for: Surgical treatment of lower urinary tract symptoms secondary to benign prostatic obstruction: an analysis and meta-synthesis of available guidelines
Source: BMC Urol. 2025 Apr 24;25:99. doi: 10.1186/s12894-025-01788-6 (PMC12020326; doi:10.1186/s12894-025-01788-6)

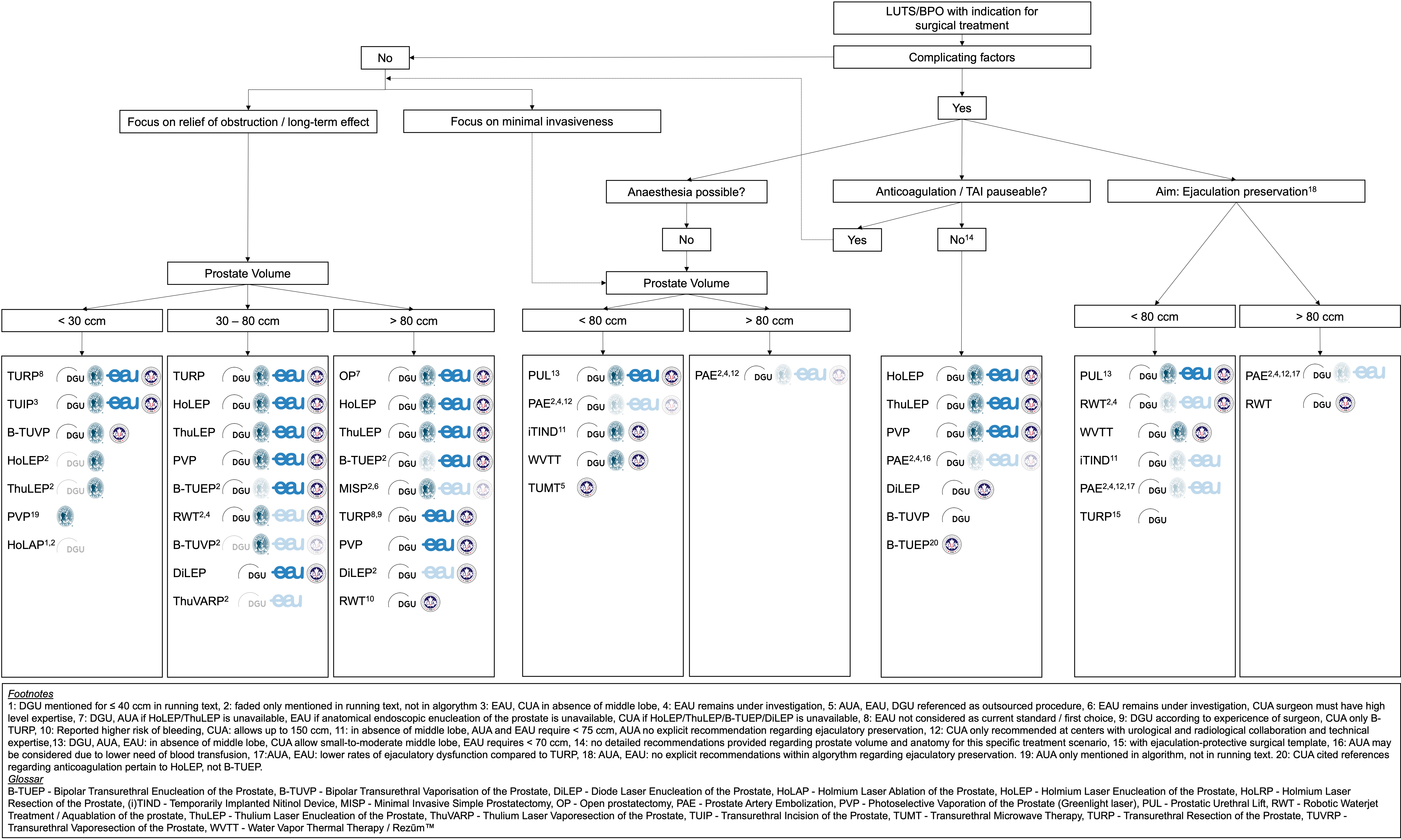

Supplement: Supplementary file 2 — Supplementary Material 2 [file 12894_2025_1788_MOESM2_ESM.jpg]
